# Test Your Memory (TYM test): diagnostic evaluation of patients with non-Alzheimer dementias

**DOI:** 10.1007/s00415-019-09447-1

**Published:** 2019-07-02

**Authors:** Jeremy Brown, Julie Wiggins, Claire J. Lansdall, Kate Dawson, Timothy Rittman, James B. Rowe

**Affiliations:** 1 Department of Neurology, Addenbrooke’s Hospital, Hills Road, Box 83, Cambridge, CB2 2QQ UK; 2 Department of Clinical Neurosciences, University of Cambridge, Cambridge, CB2 0SZ UK

**Keywords:** Dementia, Alzheimer’s, Cognitive, TYM, Non-Alzheimer dementia, Sensitivity and specificity

## Abstract

**Background/aims:**

To validate the use of the Test Your Memory (TYM) test in dementias other than Alzheimer’s disease, and to compare the TYM test to two other short cognitive tests.

**Methods:**

One hundred and fifty-seven patients with dementia other than typical Alzheimer’s disease were recruited from a specialist memory clinic. Patients completed the TYM test, the revised Addenbrooke’s Cognitive Examination (ACE-R) and Mini-Mental State Examination (MMSE), plus neurological examination, clinical diagnostics and multi-disciplinary team review. Their TYM scores were compared to age-matched controls and an Alzheimer’s disease cohort.

**Results:**

Patients scored an average of 34.4/50 on the TYM test compared to 46.0/50 in age-matched controls. Using the threshold of 42/50, the TYM test detected 80% of non-Alzheimer dementias. The area under the ROC curve was 0.89 with a PPV of 0.80 and a NPV of 0.84. The TYM test performed better than the ACE-R (using the threshold of 83) which detected 69% of cases and the MMSE (using a threshold of 24) which detected only 27%.

**Conclusions:**

The TYM test is a useful test in the detection of non-Alzheimer dementia. The TYM test performs much better than the MMSE at detecting non-Alzheimer dementias.

## Introduction

Dementia is a major health problem with the prevalence increasing as the population ages [1]. Alzheimer’s disease (AD) is the commonest form of dementia but many patients have non-Alzheimer dementias. Together, the non-Alzheimer’s diseases represent up to 50% of dementia cases [1], and are substantial causes of morbidity and mortality globally. Examples include dementia with Lewy bodies (DLB), behavioural and language variants of frontotemporal dementia (FTD), vascular dementia (VaD), progressive supranuclear palsy (PSP) and corticobasal syndrome (CBS). In addition, AD pathology can present atypically as posterior cortical atrophy (PCA) or logopaenic aphasia. The diagnosis of non-Alzheimer dementia is challenging. Patients with early dementia will normally present to non-specialists who have limited knowledge of less common dementias and the myriad ways in which they present.

The diagnosis of dementia requires a clinical assessment and examination which in most healthcare settings includes the completion of a short cognitive test (SCT) [2, 3]. It is important that any SCT used is sensitive to different forms of dementia not just AD. There has been little research into the use of SCTs in the diagnosis of non-Alzheimer dementias. The Mini-Mental State Examination (MMSE) [4] is widely used and has been extensively investigated as a diagnostic test for dementia [5] but may be insensitive to some non-Alzheimer dementias [6]. More recently devised tests such as the Addenbrooke’s Cognitive Examinations (ACE) [6, 7] and the Montreal Cognitive Assessment (MoCA) [8] are sensitive to some non-Alzheimer forms of dementia. However, both take significant time to administer and so are less used outside specialized memory clinics.

In 2009, a new SCT was published called “Test Your Memory” (TYM) [9] which was designed to test a wide range of cognitive functions using the minimum of medical time. It is self-administered by patients, under supervision, in contrast to the administration of revised Addenbrooke’s Cognitive Examination (ACE-R) and MoCA which need trained staff. The TYM test has been validated in the detection of AD in many different languages and cultures [10]. In the UK, it has been recommended by the National Institute for Health and Care Excellence as a validated cognitive assessment in non-specialist settings [3].

This study examines the properties of the TYM test in the diagnosis of non-Alzheimer dementia and compares it to the MMSE and the ACE-R.

## Methods

### Setting

Patients with non-Alzheimer’s dementia were enrolled from a UK memory clinic between May 2007 and October 2015. We aimed to recruit 20 patients with each non-Alzheimer dementia. A cross-sectional design was used to compare the TYM pattern of scoring and overall score with the clinical diagnosis. Patients were recruited prospectively, and tested during the clinical care visit. Recruitment was on a convenience basis, as NHS staff had time. Nurses administered the TYM test after the other SCTs in patients with suspected non-Alzheimer dementias. If the diagnosis was confirmed at the multi-disciplinary meeting, the patient was recruited. Data collection was complete and there were no drop outs.

Ethical approval for the study was obtained from the Cambridgeshire 2 Ethics committee.

All patients were seen by a consultant neurologist or psychiatrist with a special interest in dementia. Patients had a full clinical assessment including history, neurological examination, structural imaging (MRI scans unless there was a contra-indication), the ACE-R, MMSE as part of their clinical care, and the TYM test. Many had additional neuropsychological assessment.

Patients were diagnosed according to contemporary clinical diagnostic criteria or by consensus at a multi-disciplinary team meeting into eight different diseases: behavioural variant frontotemporal dementia (bvFTD) [11], semantic dementia (SD) [12], DLB [13], PSP [14], CBS [15, 16], progressive non-fluent aphasia/non-fluent variant primary progressive aphasia (PNFA) [12] or by consensus at a multi-disciplinary team meeting as VaD or PCA.

The clinical team had access to all the SCT scores. 87% of patients were followed up (range 2–84 months, mean 29.5 months), only the test scores at presentation was used in the analysis.

Healthy controls were age-matched and selected randomly from a previous study cohort [9]. Each group of non-Alzheimer’s dementia patients was matched with a separate control group. Controls had no cognitive complaints or history of neurological disease.

An AD cohort from an earlier study [9] with amnestic AD was used as an additional comparison group. They were of a similar age and had similar TYM scores to the non-Alzheimer’s patients (average age Alzheimer patients 69 ± 8.5 years, non-Alzheimer dementia patients 67.5 ± 7.9 years; average TYM score for AD 33.2 ± 8.2, non-Alzheimer dementia patients 34.4 ± 8.9).

### Administering the TYM test

The patient filled in the TYM test sheet under nurse supervision in a quiet room and the test was scored as described previously [9]. In contrast to the ACE-R and MMSE, the nurse supervises but does not administer the test. We used the cutoff of 42/50 for the TYM test as determined in earlier work on AD [9]. The TYM test and the scoring criteria can be downloaded free from https://www.tymtest.com.

### Statistical methods

Statistical analyses were performed in SPSSv25 and in Rv3.6.0. Group differences were assessed using independent Student’s *t* tests (equal variances not assumed) and corrected for multiple comparisons where appropriate using the Bonferroni correction. Pearson’s correlations were used to examine the relationship between the TYM, ACE-R and MMSE scores.

Cronbach’s *α* was calculated to assess the reliability of the TYM test. Data from the patients and controls were used to plot the receiver-operating characteristic (ROC) curve. Sensitivities, specificities, positive predictive value (PPV) and negative predictive value (NPV) were calculated using standard methods. The percentage of patients correctly classified in each diagnostic group were calculated.

In the text, the data are expressed as mean ± standard deviation and the *p* values refer to independent Student’s *t* test.

## Results

### Patient characteristics

One hundred and fifty-seven patients aged between 41 and 83 years were recruited. Their average age was 67.5 years. The numbers and mean ages for each of the dementia groups are shown in Table 1. There were no adverse events.

**Table 1:** Characteristics of the study patients

Diagnosis	Number of patients	Mean age and range	Sex M:F
Behavioural variant frontotemporal dementia	33	62.2 ± 8.2	21:12
Semantic dementia	24	66.9 ± 6.5	13:11
Progressive non-fluent aphasia	19	70.2 ± 7.7	9:10
Dementia with Lewy bodies	24	71.4 ± 4.8	14:10
Vascular dementia	11	71.0 ± 6.8	8:3
Progressive supranuclear palsy	22	70.4 ± 4.6	13:9
Corticobasal syndrome	17	67.7 ± 10.5	8:9
Posterior cortical atrophy	7	59.6 ± 7.4	1:6
Total	157	67.5 ± 8.0	87:70

Cronbach’s *α* was 0.75 (0.89 based on standardized items) suggesting the test was adequate to use for comparing groups.

### Combined analysis of non-Alzheimer dementia patients

The average patient TYM score was 34.4 ± 9.0, compared with 46 ± 4.6 in the age-matched controls. The overall TYM scores and all the subtests (except copying) were significantly lower than controls. The results are summarized in Table 2.

**Table 2:** TYM overall scores and subtest scores in patients with unusual dementias compared to controls

	Patients Mean ± SD	Controls Mean ± SD	*p* value
Orientation	9.2 ± 1.4	9.9 ± 0.6	< 0.001
Copy	1.7 ± 0.7	1.8 ± 0.6	0.077
Factual recall	1.8 ± 1.1	2.6 ± 0.6	< 0.001
Sums	3.0 ± 1.1	3.7 ± 0.7	< 0.001
Fluency	1.5 ± 1.5	3.4 ± 1.1	< 0.001
Similarities	2.3 ± 1.6	3.3 ± 1.1	< 0.001
Naming	4.0 ± 1.4	4.9 ± 0.4	< 0.001
Visuospatial 1	1.7 ± 1.2	2.8 ± 0.7	< 0.001
Visuospatial 2	2.8 ± 1.4	3.7 ± 0.8	< 0.001
Recall	2.2 ± 2.5	5.0 ± 1.7	< 0.001
Help	4.2 ± 1.2	5.0 ± 0.2	< 0.001
TYM score	34.4 ± 9.0	46.0 ± 4.6	< 0.001

The *p* value refers to an independent *t* test, with unequal variance, correcting for multiple comparisons across the table

**Fig. 1:**
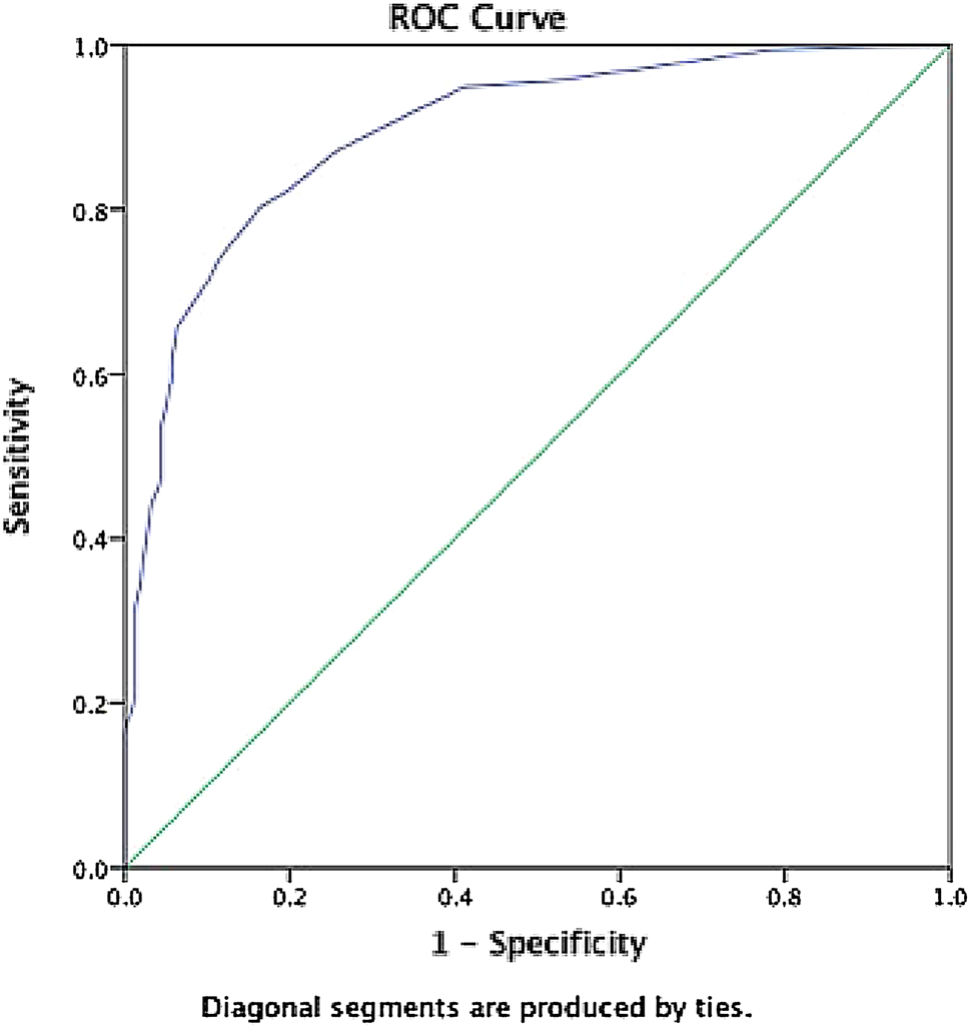
ROC curve for the TYM test in the separation of patients with non-Alzheimer dementias from normal controls

The TYM test (threshold 42) correctly identified 80% of the non-Alzheimer dementias versus controls. The area under the ROC curve was 0.89 with a PPV of 0.80 and NPV of 0.84 (Fig. 1). At the cutoff of 42, the sensitivity was 0.80 and specificity was 0.84. The non-Alzheimer dementias are a varied group of diseases, but compared to controls they scored < 50% on the verbal fluency and sentence recall subtests of the TYM.

In comparison to patients with Alzheimer’s disease, who had a similar overall score on the TYM, patients with non-Alzheimer dementias scored significantly better on orientation (*p* = 0.002), factual recall (*p* = 0.02), help given (*p* = 0.05) and recall (*p* < 0.001), had similar scores on naming, calculation and visuospatial skills but scored significantly lower on fluency (*p* = 0.01) and similarities (*p* = 0.004).

The ACE-R (threshold 82) detected 69% of patients with non-Alzheimer dementia, the MMSE (threshold 23) detected only 27%.

There were significant correlations between the TYM test and the ACE-R at the patient group level *r* = 0.83 (*p* < 0.001) and between the TYM test and the MMSE *r* = 0.69 (*p* < 0.001).

### Behavioural variant FTD

Patients with bvFTD performed significantly worse on the TYM test than controls: average 33.4 ± 10.6 vs 46.9 ± 2.4 (*p* < 0.001). The TYM test detected 73% of cases. The area under the ROC curve was 0.93.

The average bvFTD patient score on the ACE-R was 68.5 (detection rate 58%); the average score on the MMSE was 23.6 (detection rate 48%).

All bvFTD patient subtest scores were significantly lower than controls (*p* < 0.008) except copying. Patients scored less than 50% of the control score on naming and sentence recall.

**Fig. 2:**
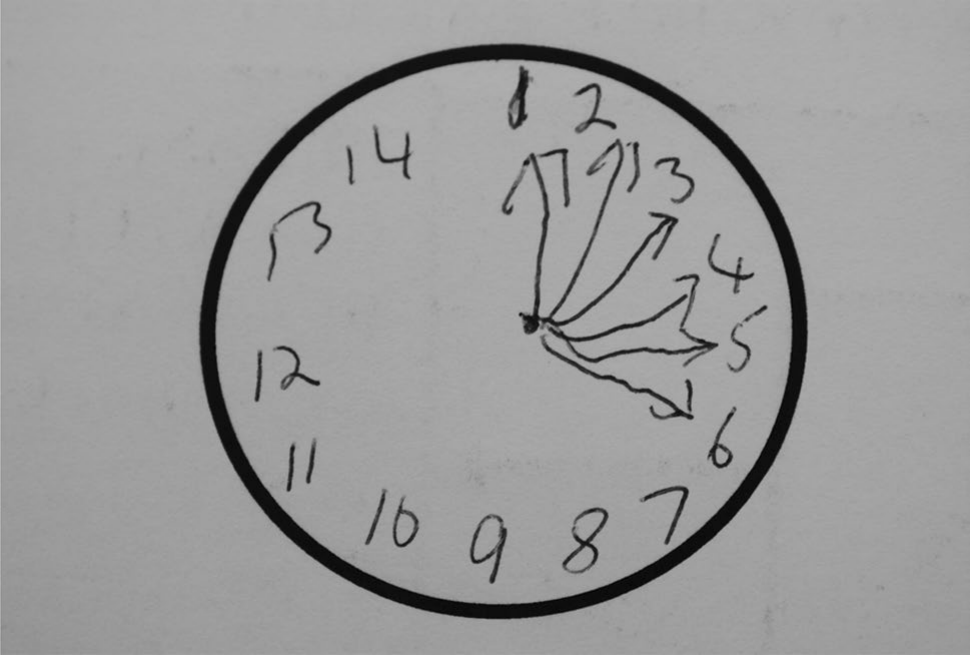
TYM test sheet filled in by a patient with bvFTD showing perseveration in drawing a clock

Perseveration of the drawing was rare but only seen in bvFTD patients (Fig. 2).

BvFTD patients scored less well on fluency and similarities and better on sentence recall than AD patients. These were not significant.

### Semantic dementia

22 patients with SD had predominantly left temporal lobe atrophy, and 2 had predominantly right-sided atrophy.

Patients performed significantly worse on the TYM test than controls: average 28.6 ± 8.2 vs 46.7 ± 3.3 (*p* < 0.001). The TYM test detected all cases of SD. The area under the ROC curve was 0.99.

The average patient score on the ACE-R was 56.6 (detection rate 96%); the average score on the MMSE was 23.5 (detection rate 46%).

All subtest scores were significantly lower than controls (*p* < 0.03) except for copying and clock drawing. SD patients scored less than 50% of the control scores on factual recall, verbal fluency, similarities, naming and sentence recall.

SD patients scored less than 50% of the AD score on fluencies (*p* < 0.001), similarities (*p* < 0.001), naming (*p* < 0.001) and sentence recall (*p* = 0.02).

### Progressive non-fluent aphasia

Patients performed significantly worse on the TYM test than controls: average 32.8 ± 8.0 vs 46.4 ± 4.1 (*p* < 0.001). The TYM test detected 89% of cases. The area under the ROC curve was 0.94.

The average patient score on the ACE-R was 69.5 (detection rate 84%); the average score on the MMSE was 24.7 (detection rate 58%).

All PNFA patient subtest scores were significantly lower than controls except orientation and copying. Patients scored less than 75% of the controls scores on factual recall, similarities, visuospatial 1 and 2, sentence recall and help.

PNFA patients scored less well on fluency and similarities but better on sentence recall than AD patients. These changes were not significant.

### Dementia with Lewy bodies

Patients with DLB performed significantly worse on the TYM test than controls: average 32.6 ± 7.6 vs 46.2 ± 6.1 (*p* < 0.001). The TYM test detected 88% of cases. The area under the ROC curve was 0.93.

The average patient score on the ACE-R was 72.8 (detection rate 88%); the average score on the MMSE was 24.9 (detection rate 21%).

**Fig. 3:**
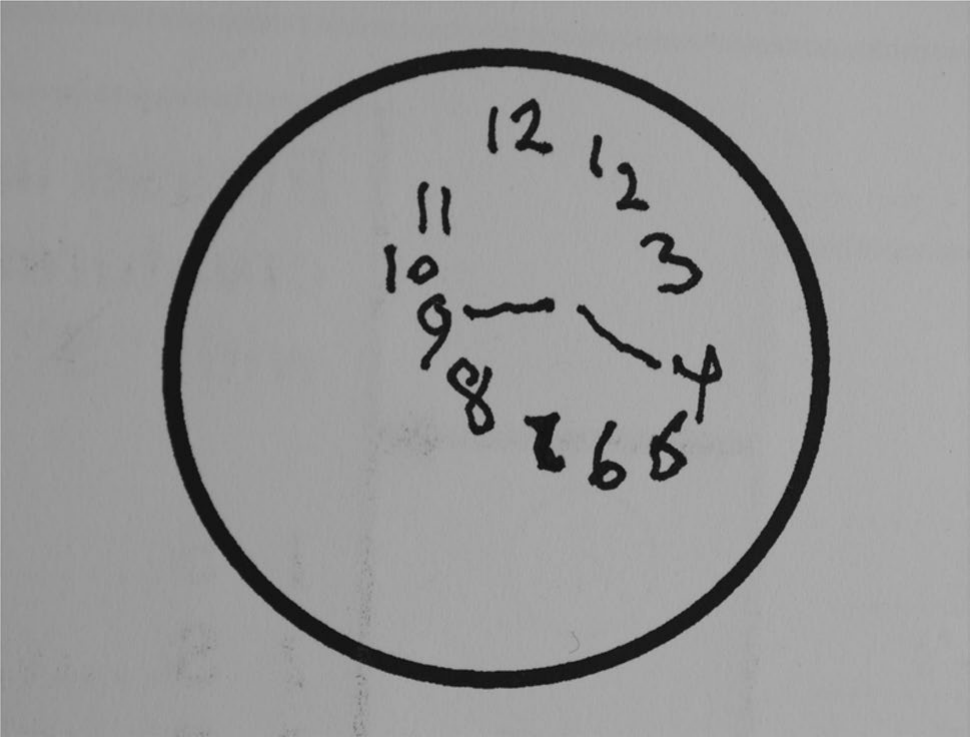
TYM test sheet filled in by a patient with DLB showing a small, poorly organized clock face

All DLB patient subtest scores were significantly lower than controls (*p* < 0.02) except for copying and similarities, they scored less than 50% of the controls scores on fluency, visuospatial 1 and sentence recall. Typically, the clock drawing was small and disorganized (Fig. 3).

DLB patients scored less well on fluency (*p* = 0.03) and better on factual and sentence recall (not significant) than AD patients.

### Vascular dementia

Patients with VaD performed significantly worse on the TYM test than controls: average 36.7 ± 8.2 vs 43.6 ± 4.8 (*p* < 0.026). The TYM test detected 73% of cases. The area under the ROC curve was 0.76.

The average patient score on the ACE-R was 76.3 (detection rate 73%); the average score on the MMSE was 25.7 (detection rate 27%).

VaD patient scores for naming and visuospatial 1 were significantly lower that controls (*p* < 0.05).

VaD patients scored better on sentence recall (not significant) than AD patients.

### Progressive supranuclear palsy

Patients with PSP performed significantly worse on the TYM test than controls: average 38.6 ± 6.5 vs 44.6 ± 5.9 (*p* = 0.002). The TYM test detected 77% of cases. The area under the ROC curve was 0.79.

The average patient score on the ACE-R was 79.7 (detection rate 59%); the average score on the MMSE was 26.5 (detection rate 18%).

PSP patients scored significantly worse than controls on sums, fluencies and visuospatial tasks (*p* < 0.009).

PSP patients scored better on orientation (*p* < 0.001), factual (*p* = 0.004) and sentence recall (*p* < 0.001) and help given (*p* = 0.01) than AD patients.

### Corticobasal syndrome

Patients with CBS performed significantly worse on the TYM test than controls: average 41.5 ± 4.5 vs 46.2 ± 6.8 (*p* < 0.024). The TYM test detected 53% of cases of CBS. The area under the ROC curve was 0.76.

The average CBS patient score on the ACE-R was 85 (detection rate 24%); the average score on the MMSE was 27.5 (detection rate 12%).

CBS patients scored significantly lower than controls on copying, fluency and visuospatial tasks (*p* < 0.05).

CBS patients scored better than AD patients on orientation (*p* < 0.001), factual (*p* < 0.001) and sentence recall (*p* < 0.09).

CBS patients scored better on sentence recall than patients with other non-Alzheimer dementias.

### Posterior cortical atrophy

PCA patients performed significantly worse on the TYM test than controls average 35.4 ± 5.0vs 46.3 ± 6.4 (*p* = 0.005). The TYM test detected 86% of cases of PCA. The area under the ROC curve was 0.88.

The average patient score on the ACE-R was 78.7 (detection rate 57%); the average score on the MMSE was 26.1 (none detected).

**Fig. 4:**
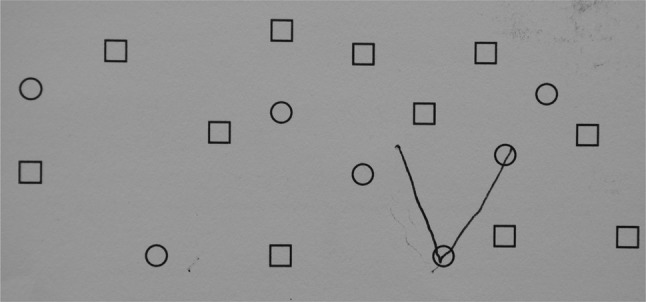
TYM test sheet filled in by a patient with PCA showing the patient not seeing the whole picture in spotting the letter in the first visuospatial task

PCA patients scored significantly worse than controls (*p* < 0.04) on sums, visuospatial tasks and help given. Visuospatial tasks were particularly poorly done and some patients showed an inability to see the whole figure (Fig. 4).

Compared to typical Alzheimer patients, PCA patients scored better on orientation (*p* = 0.002) fluency and factual/sentence recall (not significant). They scored less well on sums and visuospatial tasks (not significant).

Compared to the non-Alzheimer dementias, PCA patients scored well on fluencies but worse on sums and the visuospatial tasks.

Table 3 summarizes the TYM scores and subset scores for the different diseases.

**Table 3:** TYM scores and subtest scores for each form of unusual dementia, all non-Alzheimer dementias (including CBS and PCA), controls and patients with typical amnestic Alzheimer’s disease (AD)

	bvFTD	SD	DLB	VaD	PSP	CBS	PNFA	PCA	All non-AD	Controls	Typical AD
Orientation	8.7	9.3	8.8	9.5	9.5	9.8	9.3	9.6	9.2	9.9	8.3
Copy	1.7	1.9	1.7	1.8	1.6	1.4	1.5	1.7	1.7	1.8	1.7
Factual recall	1.8	1.1	1.9	1.8	2.1	2.5	1.4	2.0	1.8	2.6	1.4
Sums	3.1	3.3	2.7	2.8	3.0	3.2	3.0	1.7	3.0	3.7	3.1
Fluency	1.6	0.6	1.2	2	1.7	2.4	1.2	3.1	1.5	3.4	2.2
Similarities	2.0	1.0	2.5	2.9	2.8	3.4	2.1	3.1	2.3	3.3	3.0
Naming	4.0	2.2	4.3	4.1	4.5	4.8	4.6	4.4	4.0	4.9	4.4
Visuospatial 1	1.8	1.8	1.4	1.9	1.8	1.9	1.8	1.0	1.7	2.8	1.8
Visuospatial 2	3.3	3.0	2.3	2.8	2.5	3.3	2.6	1.7	2.8	3.7	2.9
Sentence Recall	1.7	0.2	1.7	2.3	4.3	4.6	1.5	3.0	2.2	5.0	0.9
Help	3.7	4.2	4.3	4.8	4.6	4.6	3.9	4.0	4.2	5.0	3.7
TYM score (0–50)	33.4	28.6	32.6	36.7	38.5	41.5	32.8	35.4	34.4	46.0	33.2
ACE-R (0–100)	68.5	56.6	72.8	76.3	79.7	85.0	69.5	78.7	72.1		66.9
MMSE (0–30)	23.6	23.5	24.9	25.7	26.5	27.5	24.7	26.1	25.1		22.5

## Discussion

The purpose of this study was to establish whether the TYM test can detect and support a clinical diagnosis of a non-Alzheimer dementia. The TYM test should not be used in isolation to diagnose dementia [17]. We compared the TYM test to 2 more established tests—the ACE-R and MMSE. Strengths of this study include the large number of patients with unusual dementias and the comparison with two groups: healthy controls and patients with typical AD.

The study has a number of limitations:

The diagnoses rested on clinical evaluation and consensus diagnostic criteria, not neuropathology. However, the TYM is intended for use in clinical settings. We recognize that some patients with CBS have Alzheimer disease pathology [16], and the majority of patients with PCA have Alzheimer’s neuropathology.

Some diagnoses changed during the study—for example a patient with PNFA later developed PSP. In these cases, the original clinical diagnosis was used. Some non-Alzheimer dementias are rare and we did not recruit equal numbers of patients in every group.

Recruitment was prospective but prolonged. A high percentage of patients were followed up for an average of 29 months. There may be ascertainment bias resulting from assessment in a specialized clinic, this is likely in VaD where patients with less typical presentations are likely to be referred to a memory clinic. The sample of VaD in this study is small and likely to be atypical.

The standardized thresholds for the SCTs were based on their performance in the diagnosis of typical AD, and alternative thresholds may improve performance for non-Alzheimer’s dementias.

This study was a proof-of-concept study. We have established that the TYM test detects a large majority of non-Alzheimer dementias. The TYM and ACE-R are both useful in detecting unusual dementias. The detection rate is dependent upon the chosen threshold and this study does not establish one as superior to the other. The ease and speed of testing is an advantage for TYM in many settings. The study showed that the TYM test and the ACE-R are superior to the MMSE in the detection of non-Alzheimer dementias.

There have been limited studies of the TYM test in non-Alzheimer dementias. A small number of patients with non-Alzheimer dementias were included in the original validation study [9]. A study recruited 47 individuals with DLB and 97 with AD in a clinic in Tokyo [18]. This study found that patients with DLB scored better than patients with AD on the memory parts of the TYM but did worse on similarities, sums and naming. These results agree with the current study, although in our study patients with DLB also did worse on fluency and the first visuospatial task.

The MMSE may be insensitive to non-Alzheimer dementias [6]. The MMSE detected just 27% of FTD cases in the original ACE validation study [6], a small study found it detected just 24% of cases of bvFTD [19]. The lack of sensitivity to non-Alzheimer’s dementias is strongly supported in this study with an overall detection rate of just 27% compared to 80% for the TYM test.

The results of the current study are in agreement with previous studies of the usefulness of the ACE in detecting FTD. In the current study, using the threshold of 83, the ACE-R detected 73% of all cases of FTD but had a lower detection rate for behavioural variant FTD (58%). If the three groups of FTD patients in this study (bvFTD, SD and PNFA) are combined then the current results are very similar to the original study ACE validation study (76.5% vs 73%) [6]. A small study [19] found that the ACE detected 83% of cases of FTD, but found that the ACE was less useful in bvFTD. A study using the ACE in semantic dementia found a very similar pattern and overall score (56.7) to the current study [20].

The ACE tests have also found to be useful in patients with PNFA [21, 22] and in the detection of cognitive deficits in patients with parkinsonian syndromes [22–24].

Fourteen patients with VaD were included in the original ACE study [6] and the ACE detected 57% of cases with a cutoff of 83 and the MMSE detected 43%. This compares to 73% and 27% in the current study.

Studies have examined the use of the MoCA in forms of VaD [25, 26] and it has been shown to identify cognitive deficits in patients post-stroke/TIA who passed the MMSE [25]. The MoCA detects cognitive deficits in patients with Parkinson’s disease dementia [26] and can detect cognitive deficits in patients with Parkinson’s disease who pass the MMSE. The MoCA had high sensitivity for detecting bvFTD from normal controls and performed better than the MMSE [27].

There is often an emphasis on the total score in interpreting short cognitive tests [17]. The total TYM score is of value if used in this way. Administering the TYM test personally and examining the pattern of scoring afterwards offers greater insights.

The TYM test detected 80% of patients with non-Alzheimer dementias. There were two main reasons for the TYM test failing to detect patients with degenerative disease. The first was that the patients had very mild cognitive problems. The lowest detection rate for the TYM test was in CBS. Many patients initially presenting with CBS have very mild cognitive problems, the 8 patients with CBS who passed the TYM test fall into this category (their average score on the ACE-R was 93/100—close to the control average of 94 [7]). This also applies to some patients with PSP, PNFA and VaD.

The second reason for passing the TYM test was that patients had focal cognitive deficits. This is illustrated by a PCA patient who just passed the TYM test scoring 43/50. However, on visuospatial tasks she scored only 4/7 (control average 6.5/7). Some patients with bvFTD just passed the TYM test but did relatively poorly on fluencies or similarities. These cases illustrate the importance of looking at the TYM sheet and the pattern of scoring not just the overall score to support a diagnosis.

In the following paragraphs, we summarize the patterns and interpretation that are useful in the different forms of dementia.

### Behavioural variant FTD

The pattern of the TYM scoring in bvFTD is distinct from Alzheimer’s disease and controls but was similar to other non-Alzheimer dementias. Patients with bvFTD scored better on memory than patients with AD but worse on fluency and similarities. The diagnosis of bvFTD can be suspected on inspection of the TYM sheet as the patient may perseverate in drawing (Fig. 2) or add comments to the sheet.

### Semantic dementia

Semantic dementia has a distinct pattern on the TYM test. The pattern of good orientation and clock drawing coupled with very poor similarities, naming, fluencies and sentence recall (due to language rather than memory problems) distinguished semantic dementia from controls, AD and other dementias.

### Progressive non fluent aphasia

Patients with PNFA scored well on memory tests compared to patients with AD. They did better on sentence recall than other dementias with the exception of patients with PSP or CBS, reflecting the clinicopathological overlap between these three disorders. They scored especially poorly on the fluency test.

### Dementia with Lewy bodies

Patients with DLB score poorly on the TYM test compared to controls. As a group, they scored poorer on fluency and visuospatial tasks compared to AD patients whilst scoring better on memory. These differences are not sufficient to allow distinction of these dementias on the TYM test alone. Compared to other non-Alzheimer dementias, patients with DLB score worse on the visuospatial tasks and sentence recall. A small disorganized clock (Fig. 2) is typical of DLB but not diagnostic.

### Vascular dementia

There was no pattern on the TYM test to distinguish VaD from other forms of dementia.

### Progressive supranuclear palsy and corticobasal syndrome

Patients with PSP and CBS often develop cognitive problems, but it is variable and may not reach the threshold for a dementia [28, 29]. As a group they scored better on the TYM test compared to the other groups. Patients scored strikingly better on sentence recall and factual recall than in other dementias, but poorly on fluencies and similarities. Some CBS patients found copying the sentence difficult.

### Posterior cortical atrophy

Patients with PCA had a distinct pattern on the TYM test which distinguished them from controls and other patients with poor scores on calculation and the visuospatial tasks with good orientation, fluency and sentence recall. Patients with severe problems found copying the sentence difficult.

In summary, the TYM test detects the majority of non-Alzheimer dementias and is superior to the MMSE in this task. The TYM requires minimal time and supervision, making it highly suited to primary and secondary healthcare settings.
